# Increased Functional Connectivity between Prefrontal Cortex and Reward System in Pathological Gambling

**DOI:** 10.1371/journal.pone.0084565

**Published:** 2013-12-19

**Authors:** Saskia Koehler, Smadar Ovadia-Caro, Elke van der Meer, Arno Villringer, Andreas Heinz, Nina Romanczuk-Seiferth, Daniel S. Margulies

**Affiliations:** 1 Berlin School of Mind and Brain and the Mind-Brain Institute, Humboldt-Universität zu Berlin, Berlin, Germany; 2 Department of Psychiatry and Psychotherapy, Charité - Universitätsmedizin Berlin, Charité Campus Mitte, Berlin, Germany; 3 Department of Psychology, Humboldt-Universität zu Berlin, Berlin, Germany; 4 Department of Neurology, Max Planck Institute for Human Cognitive and Brain Sciences, Leipzig, Germany; Hangzhou Normal University, China

## Abstract

Pathological gambling (PG) shares clinical characteristics with substance-use disorders and is thus discussed as a behavioral addiction. Recent neuroimaging studies on PG report functional changes in prefrontal structures and the mesolimbic reward system. While an imbalance between these structures has been related to addictive behavior, whether their dysfunction in PG is reflected in the interaction between them remains unclear. We addressed this question using functional connectivity resting-state fMRI in male subjects with PG and controls. Seed-based functional connectivity was computed using two regions-of-interest, based on the results of a previous voxel-based morphometry study, located in the prefrontal cortex and the mesolimbic reward system (right middle frontal gyrus and right ventral striatum). PG patients demonstrated increased connectivity from the right middle frontal gyrus to the right striatum as compared to controls, which was also positively correlated with nonplanning aspect of impulsiveness, smoking and craving scores in the PG group. Moreover, PG patients demonstrated decreased connectivity from the right middle frontal gyrus to other prefrontal areas as compared to controls. The right ventral striatum demonstrated increased connectivity to the right superior and middle frontal gyrus and left cerebellum in PG patients as compared to controls. The increased connectivity to the cerebellum was positively correlated with smoking in the PG group. Our results provide further evidence for alterations in functional connectivity in PG with increased connectivity between prefrontal regions and the reward system, similar to connectivity changes reported in substance use disorder.

## Introduction

Pathological gambling (PG) is a psychiatric disorder characterized by persistent and recurrent maladaptive gambling behavior. It is considered as a behavioral addiction since it shares clinical characteristics such as craving and loss of control with substance use disorders [1]. In the DSM-5 [2], PG has been included along with substance use disorders in the diagnostic category of ‘Substance Use and Addictive Disorders’.

A core component of addiction is diminished self-regulation, i.e. the impaired capacity to control and stop substance-taking behavior. Diminished self-regulation can be further described as a behavioral bias towards the pursuit of immediate rewards instead of the accomplishment of long-term goals [3,4]. Executive functions, which enable abdication of the immediate satisfaction of needs, have been related to the activity of the prefrontal cortex (PFC) [5]. Immediate reward seeking behavior has been linked to regions of the mesolimbic system, since subcortical areas such as the ventral striatum (including the nucleus accumbens) are highly active during reward processing [6]. Studies using functional magnetic resonance imaging (fMRI) report a functional connection between ventral striatum and medial parts of PFC [7-9]. Recently, Diekhof and Gruber [3] demonstrated a negative correlation in brain responses between the PFC and areas of the reward system (i.e., nucleus accumbens and ventral tegmental area) when subjects were in conflict between a long-term goal and an immediate reward. Furthermore, successful abdication of the immediate reward was accompanied by an increased degree of negative coupling between PFC and reward areas. Taken together, the finding of Diekhof and Gruber suggests that the ability to inhibit the behavioral bias towards immediate pleasure is related to the interaction between PFC and the reward system.

In line with the above-mentioned findings, fMRI studies found functional alterations in the PFC as well as in the mesolimbic system in substance dependence. Drug-addicted individuals show a PFC dysfunction with a related decrease in performance during executive function tasks [10]. Within the reward system, an excessive sensitivity (i.e., enhanced brain responses) to drug-related stimuli [11-13] and reduced brain activity to non-drug rewards [13-16] has been described in individuals with alcohol and nicotine dependence, and increased brain activity in response to non-drug rewards has been found in individuals with cocaine dependence [17]. Taking these alterations into account, an imbalance between prefrontal brain activity and mesolimbic function has been suggested to contribute to addictive behavior [18,19]. 

Functional changes in the PFC and mesolimbic reward system have also been reported in PG. Patients with PG have demonstrated decreased ventromedial prefrontal activation during an inhibition task [20], which indicates a frontal lobe dysfunction, and is in line with previous behavioral studies on executive function and decision-making in PG [21-24]. Moreover, PG patients displayed decreased prefrontal activation when obtaining monetary reward [25-27], and increased dorsolateral prefrontal activation in response to videos and pictures with gambling scenes [28,29], suggesting changes in the processing of reward-indicating stimuli. Accordingly, studies using event-related potentials suggest a medial frontal hypersensitivity to reward in problem gamblers [30,31]. Alterations in reward processing have also been found in the ventral striatum: PG patients showed blunted activation during anticipation of monetary reward [25,32], whereas increased activity was reported for problem gamblers [33]. PG patients also demonstrated decreased activation when obtaining a monetary reward [27], and an increased activation in response to pictures with gambling scenes [29], indicating altered brain responses within the reward system for gambling-related stimuli. These findings suggest that PG patients show dysfunctional changes independently in prefrontal as well as mesolimbic brain structures.

The functional interaction between the prefrontal and mesolimbic system can be explored using resting-state functional connectivity – i.e., the temporal correlation of spontaneous blood oxygenation level-dependent (BOLD) fMRI signal between brain areas. Patterns of intrinsic functional connectivity are correlated with similar patterns to those activated during tasks-related activity [34,35]. Resting-state fMRI has the additional advantage for a clinical population of not requiring task performance and a relatively short scanning duration (< 10 minutes) [36]. Recently, resting-state fMRI studies reported changes in functional connectivity in substance use disorders [37-47]. Some of these studies suggest patterns of altered connectivity between cognitive control nodes such as lateral PFC, anterior cingulate cortex and parietal areas [39,41,46], and alterations in connectivity from the ventral striatum [38,41,43-45] with mixed results regarding the connectivity patterns of PFC and ventral striatum. Increased functional connectivity between ventral striatum and orbitofrontal PFC was found in chronic heroin users [41]. In contrast, another study with opioid dependent individuals [44] observed reduced functional connectivity between nucleus accumbens and orbitofrontal PFC. Moreover, studies on cocaine abuse / dependence demonstrated increased functional connectivity between ventral striatum and ventromedial PFC [45] and reduced prefrontal interhemispheric connectivity [39]. Together, these resting-state studies demonstrate that the interaction between PFC and the mesolimbic reward system is altered in patients with substance use disorders.

To date, little is know about functional connectivity alterations in a behavioral addiction such as PG. A first indication for an altered fronto-striatal functional connectivity in PG was found in an exploratory resting-state study by Tschernegg et al. [48]. By using a graph-theoretical approach, they observed increased functional connectivity between caudate and anterior cingulate in PG patients as compared to controls. However, it remains unclear whether PG patients demonstrate similar alterations in the interaction between PFC and the core structure of the reward system (i.e., ventral striatum) as reflected by functional connectivity findings in substance-related addictions. To the best of our knowledge, no such study on PG has yet been published. Therefore, the present study examines patterns of functional connectivity in the prefrontal and the mesolimbic system in patients with symptoms of PG. Functional connectivity analysis was based on externally defined regions-of-interests (“seeds”) located in the middle frontal gyrus and ventral striatum, which were based on the results of a previous voxel-based morphometry (VBM) study [49]. Since activation studies of PG found an association between symptom severity [27] as well as impulsiveness [25] and evidence of brain functional alteration, we assumed that these behavioral measures as well as smoking behavior as an additional marker for addictive behavior would be related to functional alteration of the relevant networks in the PG group.

## Materials and Methods

### Ethics Statement

The study was performed in accordance with the Declaration of Helsinki and approved by the Ethics Committee of the Charité - Universitätsmedizin Berlin. All participants gave written informed consent prior to participation.

### Participants

Data from 19 PG patients (mean age 32.79 years ± 9.85) and 19 controls (mean age 37.05 years ± 10.19), who participated in an fMRI study at the Charité - Universitätsmedizin Berlin (see Supplementary Methods in File S1), were used for resting-state fMRI analysis. PG patients were recruited through Internet advertisement and notices in casinos. They were neither in an abstinent state nor treatment seeking. Diagnosis for PG was based on a German questionnaire for gambling behavior (“Kurzfragebogen zum Glücksspielverhalten”, KFG) [50]. The questionnaire contains 20 items and is based on the DSM-IV / ICD-10 diagnosis criteria for PG. The cut-off for PG is set to 16 points. We also applied the Gambling Symptom Assessment Scale (G-SAS) [51] as an additional measure of symptom severity. None of the PG patients or controls had a known history of any neurological disorder or current psychiatric Axis-I disorder including drug or alcohol dependence as verified by an interview according to the Structured Clinical Interview for DSM-IV Axis I Disorder (SCID-I) [52]. Controls did not show any severe gambling symptoms as confirmed by the KFG. 

Handedness was measured by the Edinburgh Handedness Inventory [53]. We collected information about years of school education, number of cigarettes per day, alcohol per month in grams, and fluid intelligence assessed with the matrices test of the Wechsler Intelligence test for adults [54]. Smokers were not allowed to smoke for 30 minutes prior to the scan session. 

Impulsiveness was measured using the German version of the Barratt Impulsiveness Scale-Version 10 (BIS-10) [55], which contains 34 items subdivided into three impulsiveness subscores: nonplanning, motor and cognitive impulsiveness. After the fMRI scan, the desire for gambling (craving) was measured by a visual analog scale (VAS), in which participants answered five craving-related questions (e.g., ”How strong is your intention to gamble?”) by marking a line between a 0 (‘‘not at all’’) to 100 % (‘‘extremely strong’’).

For the functional connectivity analysis of the middle frontal seed region, all 38 subjects were analyzed. Groups did not differ in education, fluid intelligence, smoking habits, alcohol intake nor handedness (Table 1). In terms of gambling habits, 17 PG patients mainly used slot machines and two PG patients were bettors.

**Table 1:** Socio-demographic, clinical and psychometric data for the whole sample and for the subsample used for ventral striatal seed analysis.

	PG patients (N = 19)	controls (N = 19)			PG patients (N = 14)	controls (N = 18)		
	Mean (SD)	Mean (SD)	*t*-value	*p*-value	Mean (SD)	Mean (SD)	*t*-value	*p*-value
age in years	32.79 (9.85)	37.05 (10.19)	1.31	.20	31.29 (9.09)	36.50 (10.19)	1.50	.14
number of cigarettes per day	5.11 (7.23)	6.79 (8.39)	0.66	.51	5.43 (8.15)	6.06 (7.98)	0.22	.83
alcohol intake in grams	128.74 (210.89)	161.19 (184.38)^1^	0.50	.62	153.00 (236.28)	167.74 (187.89)^2^	0.19	.85
years of school education	10.82 (1.95)	11.32 (1.57)	0.87	.39	11.32 (1.75)	11.39 (1.58)	0.11	.91
fluid intelligence (matrices test)	17.42 (4.22)	19.21 (3.66)	1.40	.17	18.36 (3.69)	19.17 (3.76)	0.61	.55
handedness (EHI)	65.34 (66.60)	81.03 (38.19)	0.89	.38	54.39 (75.01)	82.90 (38.39)	1.40	.17
BIS-10 total	2.38 (0.41)	1.96 (0.27)	3.73	.001	2.42 (0.44)	1.97 (0.27)	3.54	.001
BIS-10 cognitive	2.30 (0.39)	1.85 (0.33)	3.88	< .001	2.34 (0.45)	1.86 (0.34)	3.49	.002
BIS-10 motor	2.33 (0.56)	1.86 (0.36)	3.08	.004	2.38 (0.55)	1.85 (0.36)	3.31	.002
BIS-10 nonplanning	2.52 (0.38)	2.18 (0.38)	2.76	.009	2.54 (0.38)	2.21 (0.35)	2.48	.019
KFG	32.95 (10.23)	1.42 (2.32)	13.10	< .001	34.21 (10.81)	1.50 (2.36)	12.52	< .001
G-SAS	21.05 (9.37)	1.94 (2.90)^1^	8.28	< .001	22.14 (10.11)	2.00 (2.98)^2^	7.84	< .001
VAS craving in %	34.62 (29.80)	17.19 (16.77)	2.22	.033	33.41 (29.32)	16.97 (17.23)	1.99	.056

Note: Two sample *t*-test (two-tailed) with *df* = 36 (**^*1*^**N_controls_ = 18, *df* = 35) for the whole sample and *df* = 30 (**^*2*^**N_controls_ = 17, *df* = 29) for the subsample. EHI, Edinburgh Handedness Inventory; BIS-10, Barratt Impulsiveness Scale-Version 10; KFG, “Kurzfragebogen zum Glücksspielverhalten” (gambling questionnaire); G-SAS, Gambling Symptom Assessment Scale; VAS, visual analog scale.

For the functional connectivity analysis of the ventral striatal seed region, we had to exclude five PG patients and one control subject due to lack of complete brain coverage in that area (see *fMRI data analysis*); these subgroups consist of 14 PG patients (mean age 31.29 years ± 9.09) and 18 controls (mean age 36.50 years ± 10.19). Groups did not differ in education, fluid intelligence, smoking habits, alcohol intake nor handedness (Table 1). Thirteen PG patients mainly used slot machines and one PG patient was bettor.

### MRI acquisition

Imaging was performed on a 3 Tesla Siemens Magnetom Tim Trio (Siemens, Erlangen, Germany) at the Charité - Universitätsmedizin Berlin, Campus Benjamin Franklin, Berlin, Germany. For the functional imaging session, the following scanning parameters were used: repetition time (TR) = 2500 ms, echo time (TE) = 35 ms, flip = 80°, matrix = 64 * 64, field of view (FOV) = 224 mm, voxel size = 3.5 * 3.5 * 3.0, 39 slices, 120 volumes. 

For the purpose of anatomical registration of the functional data, we acquired an anatomical scan using a three-dimensional magnetization prepared rapid gradient echo (3D MPRAGE) with the following parameters: TR = 1570 ms, TE = 2.74 ms, flip = 15°, matrix = 256 * 256, FOV = 256 mm, voxel size = 1 * 1 * 1 mm^3^, 176 slices.

### fMRI data analysis

Images were preprocessed and analyzed using both FMRIB Software Library (FSL, http://www.fmrib.ax.ac.uk/fsl) and Analysis of Functional Neuroimages (AFNI, http://afni.nimh.nih.gov/afni/). Preprocessing was based on the 1000 Functional Connectomes scripts (www.nitrc.org/projects/fcon_1000). The following preprocessing steps were performed: slice-time correction, motion correction, spatial smoothing with a 6 mm full-width at half maximum Gaussian spatial filter, band pass filtering (0.009 - 0.1 Hz) and normalization to the 2 * 2 * 2 mm^3^ Montreal Neurological Institute (MNI)-152 brain template. Signal from regions of no-interest: white matter and cerebrospinal fluid signal were removed using regression. Global signal was not removed as it has recently been shown that this preprocessing step can induce false-positive group differences [56].

**Figure 1:**
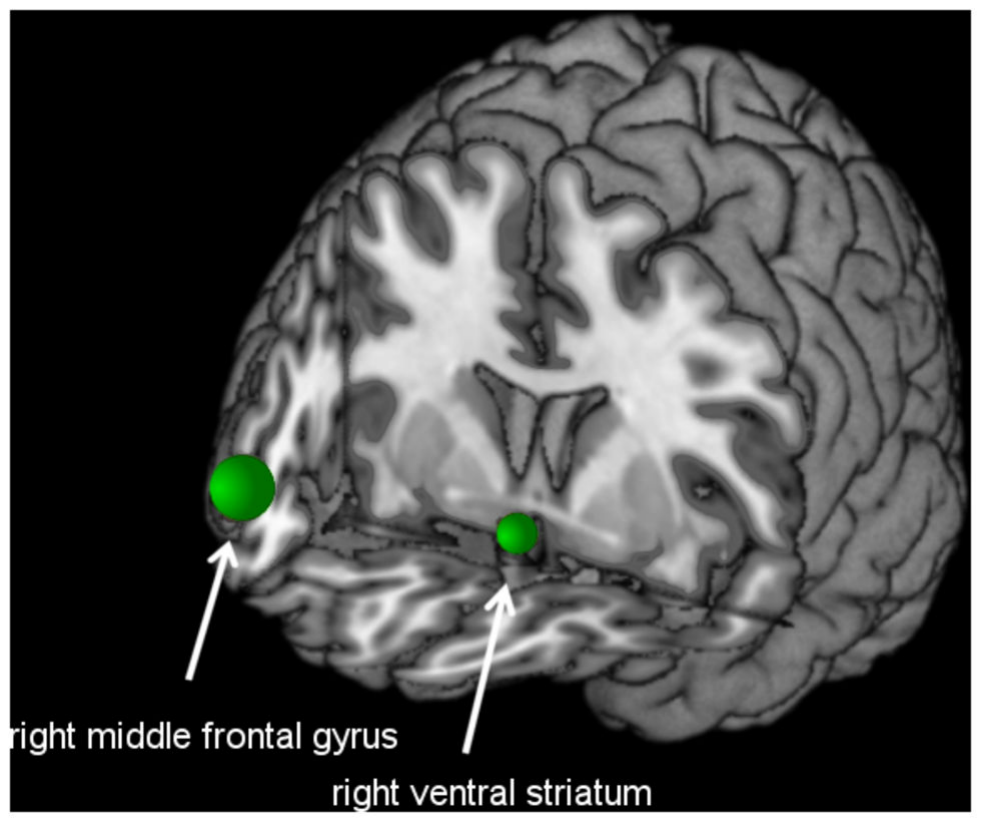
Location of seed regions for functional connectivity analysis Right middle frontal gyrus: x = 44, y = 48, z = 7, radius of 6 mm. Right ventral striatal seed: x = 5, y = 6, z = -12, radius of 4 mm.

Seed regions for functional connectivity analysis were defined based on the results of a previous VBM study using the participants' structural data from the current study [49]. In this study, PG patients demonstrated an increase in local gray matter centered in right middle frontal gyrus (x = 44, y = 48, z = 7, 945 mm^3^) and right ventral striatum (x = 5, y = 6, z = -12, 135 mm^3^). In the functional connectivity analysis, spheres were defined at the peak points of the gray matter differences (Figure 1). Sphere radii were chosen such that the significant area from the VBM analysis would correspond to the size of the sphere. For the prefrontal seed, we used a radius of 6 mm (880 mm^3^, 110 voxels). For the ventral striatal seed, we used a radius of 4 mm (224 mm^3^, 28 voxels). Due to signal loss in the orbitofrontal cortex and adjacent subcortical structures we had to exclude six subjects from the functional connectivity analysis for the ventral striatal seed (Figure S1). A subject was excluded if there were less than 50% of voxels within the seed region. 

We conducted a voxel-wise functional connectivity analysis for each seed region. Averaged time courses were extracted from each seed region for each subject, and linear correlation coefficients between the seed region time course and the time course for all other voxels in the brain was computed using the 3dFIM+ AFNI command. Correlation coefficients were then transformed to *z*-values using the Fisher *r*-to-*z* transformation. The *z*-values were used for the within and between group analyses. For each group, one-sample *t*-tests were carried out for each seed region in order to provide correlation maps within each group. Group comparisons for each seed region were performed using two-sample *t*-tests. To account for gray matter-related differences in functional connectivity, which might be due to using seed regions based on the VBM results, we used the individual gray matter volume as a voxel-wise covariate (see Supplementary Results in File S1 and Table S1 for the results of the functional connectivity analysis without gray matter regression, and Figure S2 and Figure S3 for an illustration of both the analysis with and the analysis without gray matter regression). Group level results for connectivity maps were thresholded at a *z*-score > 2.3, corresponding to *p* < .01. To account for the problem of multiple comparisons, we performed a cluster-wise correction using Gaussian random field theory implemented in FSL, and a Bonferroni correction for the number of seeds.

In order to examine whether changes in functional connectivity within the PG group were related to impulsivity, symptom severity and smoking habits, we extracted the mean *z*-value for the significant, thresholded clusters (two clusters for right middle frontal seed and two clusters for right ventral striatal seed) for each of the PG patients. Then, the *z*-values were correlated with the self-report measures of interest (BIS-10 total and subscores, KFG, G-SAS, VAS craving, number of cigarettes per day). 

Finally, we tested for the correlation between both seeds for the subsample by computing the Pearson’s correlation between the extracted time courses.

### Behavioral data analysis

Clinical, socio-demographic and psychometric data, as well as the association between *z*-values and self-report measures of interest, were analyzed using SPSS Statistics 19 (IBM Corporation, Armonk, NY, USA). Group comparisons were carried out using two-sample *t*-test (two-tailed). Correlations were computed using the Pearson’s and Spearman’s correlation coefficients. An alpha error probability of < .05 was used.

## Results

### Clinical and psychometric data

We Found Significantly Higher Scores for Gambling Severity (KFG, G-SAS), Craving for Gambling (VAS) and Impulsiveness (BIS-10) in PG Patients as Compared to Controls (Table 1). 

### Connectivity from the right middle frontal gyrus (N_controls_ = 19, N_PGpatients_ = 19)

**Figure 2:**
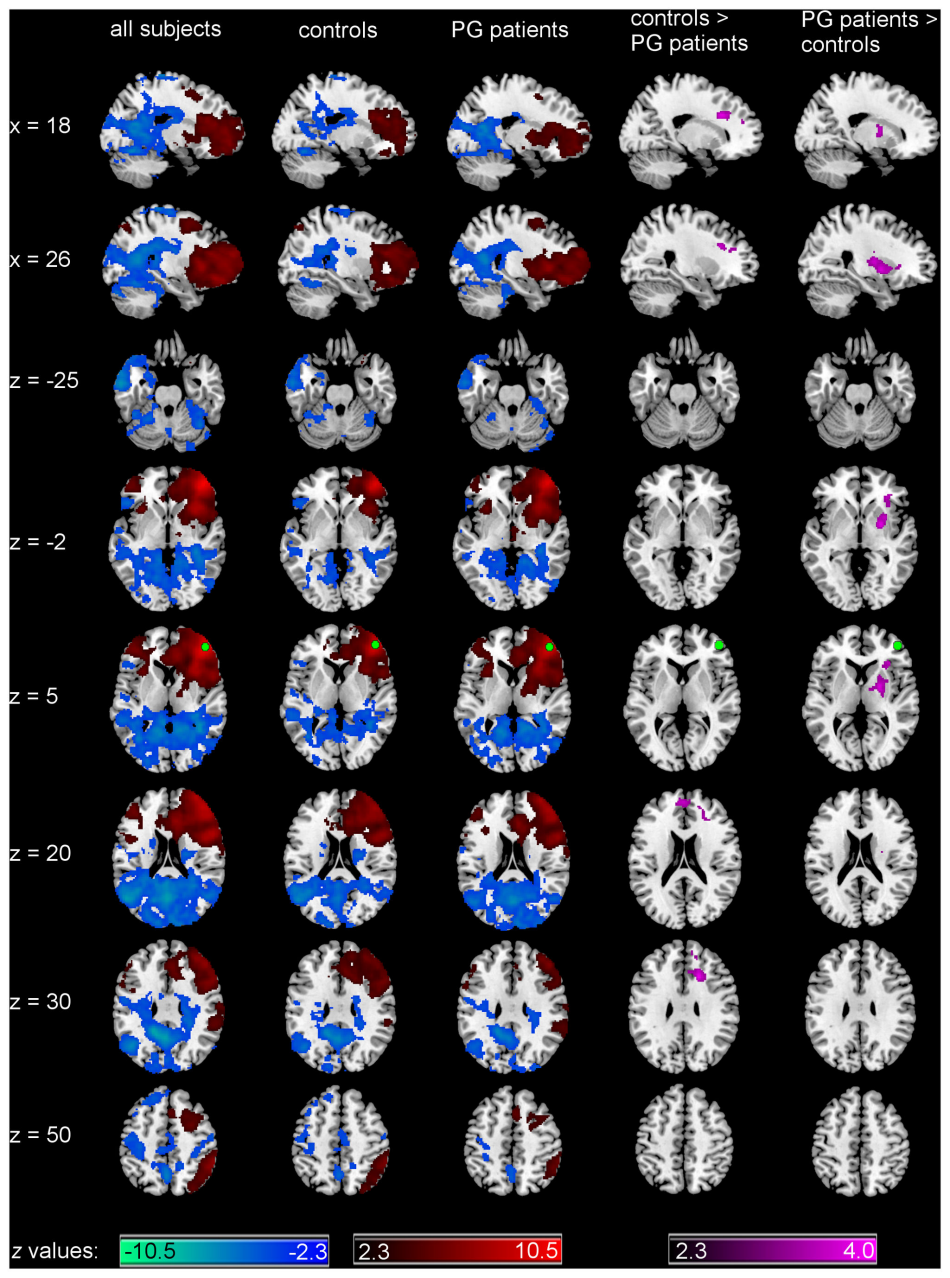
Functional connectivity of right middle frontal seed Patterns of significantly positive (red spectrum) and negative (blue spectrum) correlations with the right middle frontal gyrus (seed depicted in green) within all subjects and within the groups. Group comparison for significant correlations: PG patients < controls and PG patients > controls (violet spectrum). All maps are thresholded at a *z*-score > |2.3| (cluster-wise corrected using Gaussian random field theory and Bonferroni corrected for the number of seeds). N_controls_ = 19, N_PGpatients_ = 19.

Across both groups (Figure 2 and Table 2), maximal connectivity from the right middle frontal gyrus was found to the right hemisphere around the seed, which extended to the right PFC as well as right insula, striatum, angular gyrus, lateral occipital cortex and supramarginal gyrus. Moreover, significant positive connectivity from the right middle frontal gyrus was found to its contralateral homologue region (left lateral PFC) extending to the left insula. Negative connectivity was found to the left posterior cingulate gyrus extending to left temporal pole, and regions in both hemispheres such as lingual gyrus, intracalcarine cortex, occipital pole, precuneus, pre- and postcentral gyrus, superior frontal gyrus, thalamus, bilateral cingulate gyrus, and cerebellum.

**Table 2:** Brain regions exhibiting significant connectivity across both groups and for the group contrasts.

Seed	Contrast	Anatomical region	Side	Cluster-level *p*-value (corrected)	Cluster size(voxels)	Voxel-level *z*-value	MNI coordinates at peak voxel		
							x	y	z
Right middle frontal gyrus	mean positive	frontal pole	R	< .0001	26241	10.4	46	48	10
	mean negative	posterior cingulate gyrus	L	< .0001	50437	7.18	-14	-50	32
	PG < controls	cingulate gyrus	R	.0015	508	3.65	18	20	30
	PG > controls	putamen	R	.0026	668	3.47	26	0	-2
Right ventral striatum	mean positive	nucleus accumbens	R	< .0001	9025	8.93	8	6	-10
	mean negative	precentral gyrus	L	< .0001	17987	5.22	-50	2	20
		lingual gyrus	L	< .0001	2362	4.7	-10	-80	-12
	PG < controls			not significant					
	PG > controls	cerebellum	L	.0026	670	4.31	-32	-52	-38
		superior frontal gyrus	R	.0101	543	3.92	26	26	50

Note: Two sample *t*-test (two-tailed) with *df* = 36 (**^*1*^**N_controls_ = 18, *df* = 35) for the whole sample and *df* = 30 (**^*2*^**N_controls_ = 17, *df* = 29) for the subsample. EHI, Edinburgh Handedness Inventory; BIS-10, Barratt Impulsiveness Scale-Version 10; KFG, “Kurzfragebogen zum Glücksspielverhalten” (gambling questionnaire); G-SAS, Gambling Symptom Assessment Scale; VAS, visual analog scale.

**Figure 3:**
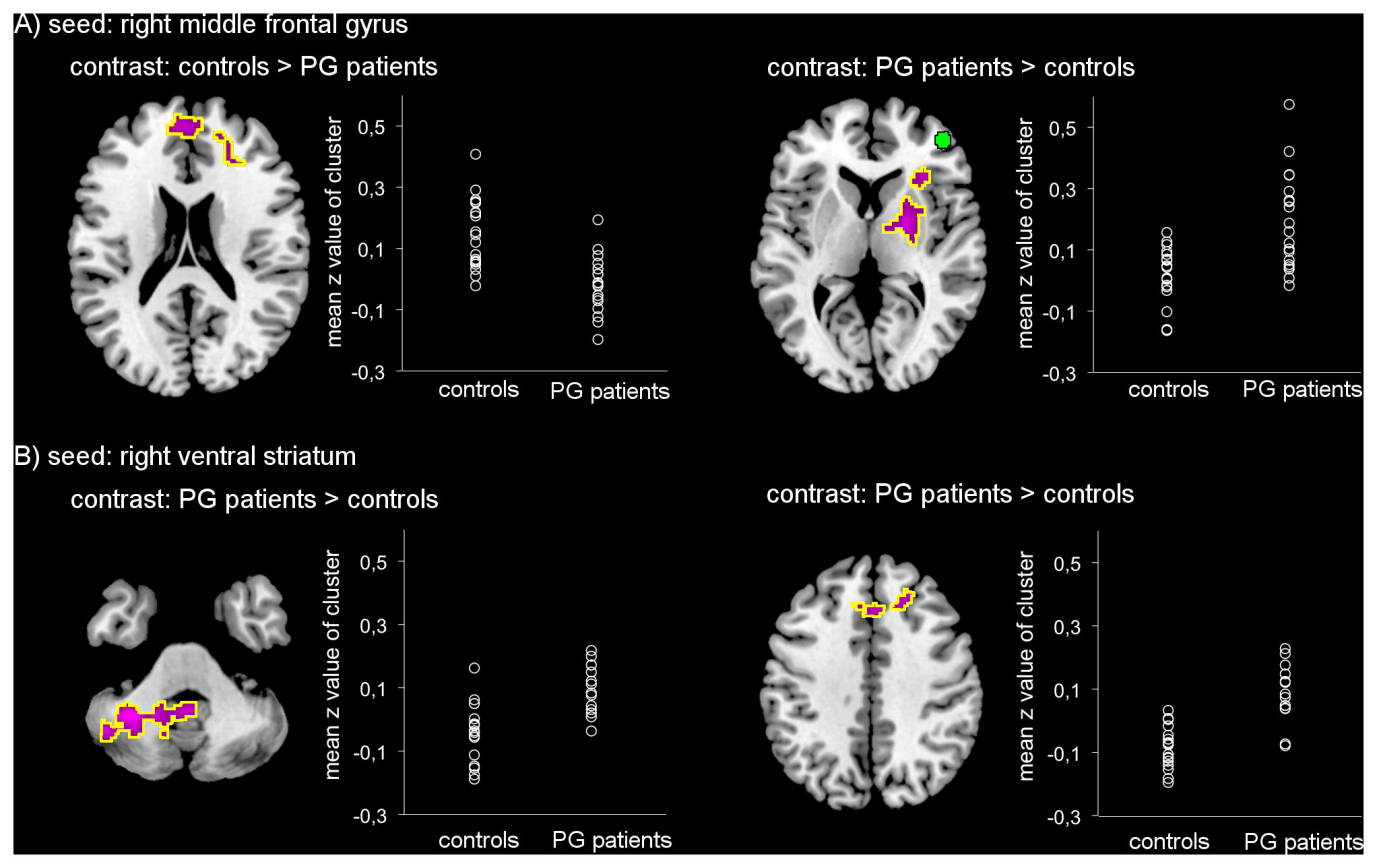
Group differences in functional connectivity of the seeds Plots show *z*-values for the significant clusters of difference (encircled in yellow). Number of subjects for right middle frontal gyrus seed region A): N_controls_ = 19, N_PGpatients_ = 19, and for right ventral striatal seed region B): N_controls_ = 18, N_PGpatients_ = 14.

Group contrasts (Figure 2, Figure 3A and Table 2) revealed increased connectivity from the right middle frontal gyrus to the right striatum for PG patients as compared to controls. The peak voxel of this contrast is in the putamen with the cluster extending into the globus pallidus, dorsal caudate, insula and thalamus. Decreased connectivity was found to the right anterior cingulate cortex extending to the bilateral superior frontal and paracingulate gyrus in PG patients as compared to controls. 

The group differences remained consistent using subgroups that included only individuals with full striatal coverage (N_controls_ = 18, N_PGpatients_ = 14; results not shown).

Connectivity from the right ventral striatum (N_controls_ = 18, N_PGpatients_ = 14)

**Figure 4:**
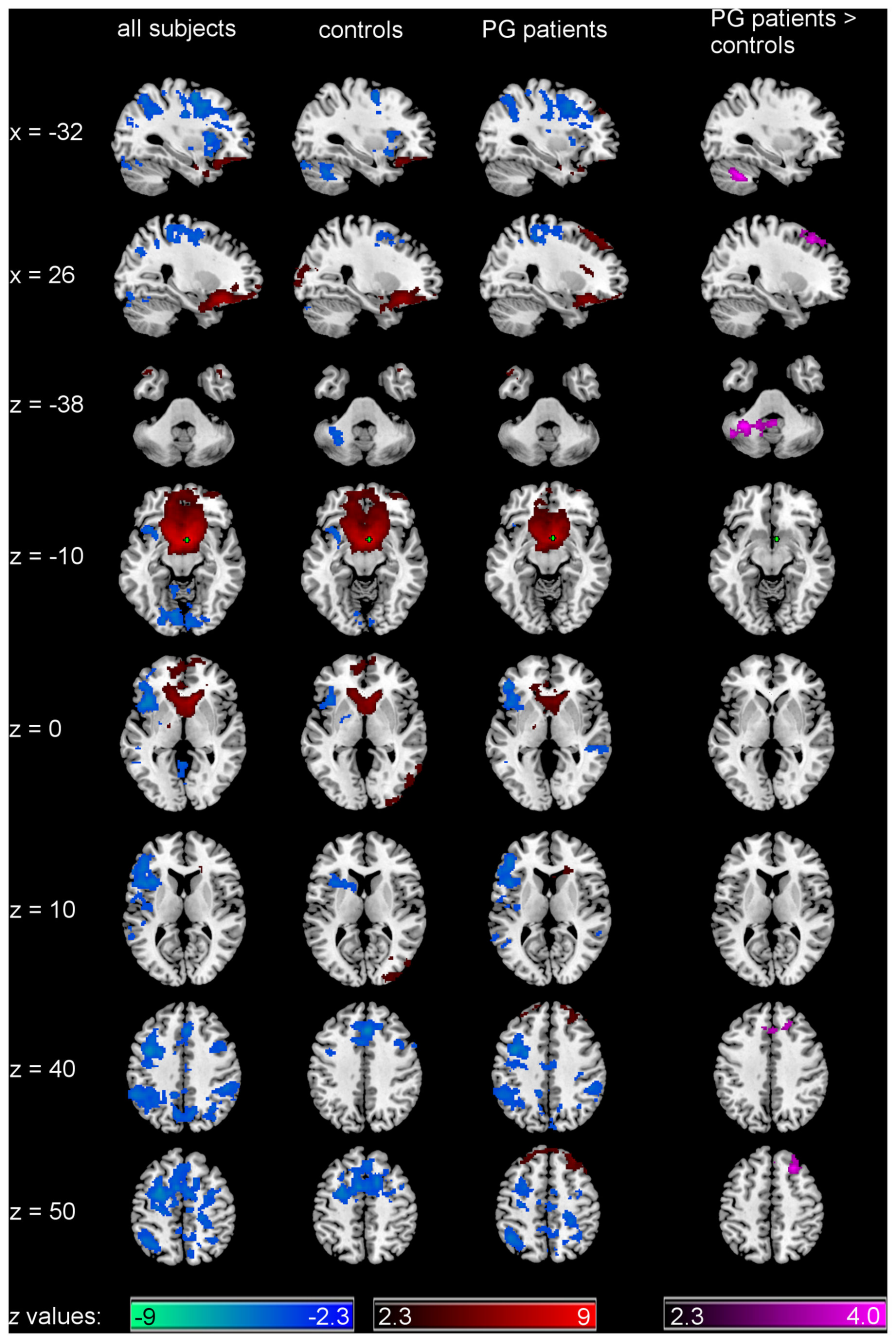
Functional connectivity of right ventral striatal seed Patterns of significantly positive (red spectrum) and negative (blue spectrum) correlations with the right ventral striatum (seed depicted in green) within all subjects and within the groups. Group comparison for significant correlations: PG patients > controls (violet spectrum). Please note that the contrast controls > PG patients was not significant. All maps are thresholded at a *z*-score > |2.3| (cluster-wise corrected using Gaussian random field theory and Bonferroni corrected for the number of seeds). N_controls_ = 18, N_PGpatients_ = 14.

Across both groups (Figure 4 and Table 2), the maximal connectivity from the right ventral striatum was found surrounding the seed and in the contralateral homologue region, including bilateral nucleus accumbens and subcallosal gyrus, and extending to bilateral caudate, putamen, amygdala, ventromedial PFC, and the frontal and temporal poles. Negative connectivity was found in the right precentral gyrus extending to bilateral paracingulate, middle frontal, inferior frontal and superior frontal gyrus, right postcentral gyrus, and left hemispheric areas such as frontal pole, insula and the frontal and central operculum. Negative connectivity was also found in the left lingual gyrus extending to the right lingual gyrus and regions in bilateral cerebellum, and bilateral occipital fusiform gyrus, and in the bilateral supramarginal gyrus extending to superior parietal lobule, bilateral lateral occipital cortex, precuneus and angular gyrus. 

Group contrasts (Figure 4, Figure 3B and Table 2) revealed increased connectivity from the right ventral striatum to the left cerebellum as well as to the right superior frontal gyrus, extending to the right middle frontal gyrus and bilateral paracingulate gyrus in PG patients as compared to controls. 

### Correlation with self-report measures

**Figure 5:**
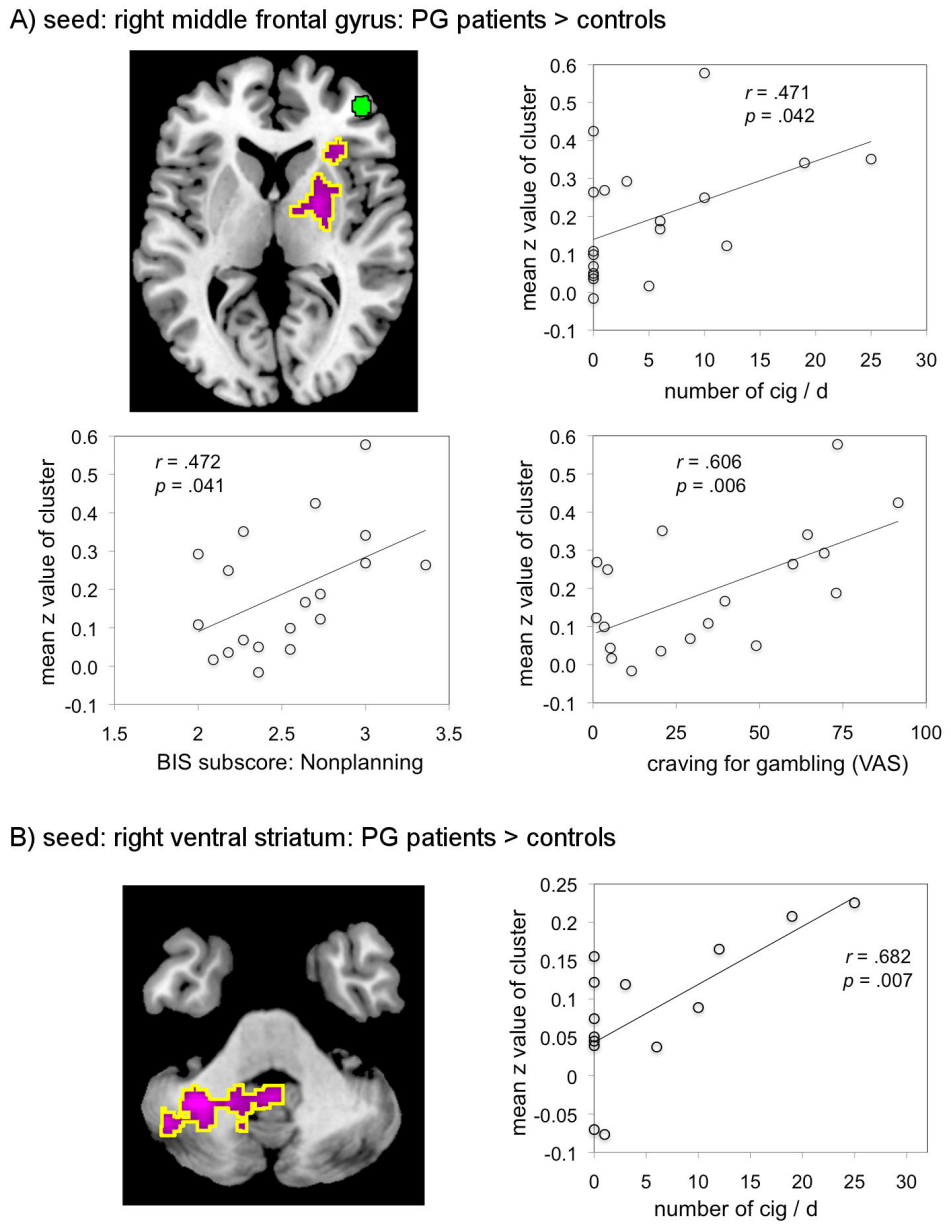
Significant positive correlations for connectivity patterns Scatter plots show significant correlations between the mean *z*-values of the thresholded clusters of the group contrasts PG patients > controls and smoking habits (number of cigarettes per day [cig/d]), the nonplanning BIS subscale and the VAS for craving. Number of PG patients for right middle frontal gyrus seed region A): N_PGpatients_ = 19, and for right ventral striatal seed region B): N_PGpatients_= 14.

The mean *z*-values in clusters of significant difference between the two groups were used to test for correlations with behavioral measures within the PG group (4 clusters). Positive correlations were found for connectivity between the right middle frontal seed and the striatum (for the PG > controls contrast) and the nonplanning BIS-10 subscale, smoking habits (number of cigarettes per day) and craving scores (Figure 5A). We also found a positive correlation for connectivity between the right ventral striatal seed and cerebellum (for the PG > controls contrast) and smoking habits (Figure 5B). Since smoking habits were not normally distributed, we also computed Spearman’s correlation coefficient for this variable. For the right middle frontal seed mean *z*-score the correlation was still significant, *rS* = .52, *p* = .021. For the right ventral striatal seed mean *z*-score, we got a marginal significant result, *rS* = .51, *p* = .06. We did not find any significant correlation for the other BIS-10 subscales and BIS-10 total and for KFG and G-SAS. 

### Correlation between the right middle frontal gyrus and right ventral striatum (N_controls_ = 18, N_PGpatients_ = 14)

Groups did not significantly differ in the correlation values between the prefrontal and ventral striatal seeds.

## Discussion

We found that PG patients demonstrate increased functional connectivity between regions of the PFC and mesolimbic reward system, as well as reduced connectivity in the area of the PFC. Specifically, PG patients demonstrated increased connectivity between the right middle frontal gyrus and the right striatum as compared to controls, which was positively correlated with the nonplanning BIS subscale, smoking and craving scores. Reduction in connectivity was found in PG patients from the right middle frontal gyrus to other prefrontal areas. Importantly, on the group level we observed functional connectivity from the ventral striatum to parts of the orbital PFC, which replicate previously reported connectivity patterns [7,8,57]. 

An imbalance between prefrontal function and the mesolimbic reward system has been suggested to contribute to addictive behavior [18,19] based on studies in patients reporting altered function of the PFC [10], as well as functional changes in areas of the reward system such as the ventral striatum [11-16]. Similar to our finding of an increased functional connectivity between PFC and striatum, Tschernegg et al. [48] observed increased fronto-striatal functional connectivity in PG patients as compared to controls using a graph-theoretical approach. Altered intrinsic functional connectivity between the PFC and the reward system was also reported for substance use disorder [41,44,45,58]. An increased connectivity between the ventromedial / orbitofrontal PFC and ventral striatum has been found in chronic heroin users [41] and abstinent cocaine users [45]. The altered interaction between prefrontal structures and the mesolimbic reward system in PG shares similar functional organization to these substance-related addictions, suggesting a more general pathomechanism for disorders related to an increase in habitual pathological behavior. 

In addition, we found a decrease in functional connectivity between the right middle frontal gyrus and other prefrontal areas (i.e., right anterior cingulate cortex extending to the bilateral superior frontal and paracingulate gyrus) in PG patients as compared to controls. Together with the results of imaging and behavioral studies on PG that report diminished ventromedial PFC activity [20,59] and impaired executive function and decision-making [21-24], our finding suggests an alteration in the functional organization of the PFC. However, we did not find any differences between PG patients and controls for fluid intelligence, a construct which has been associated with frontal lobe function [60], suggesting that the observed alteration in connectivity does not effect overall cognitive capacity, and may rather be specific to the underlying disease process. Altered connectivity within the PFC is in line with prefrontal abnormalities reported in task activation [10] and resting-state fMRI studies on substance use disorder [39,41] and PG [48]. Moreover, it might contribute to the altered interaction between PFC and a core area of the brain reward system, the ventral striatum, and may influence prefrontal top-down modulation of reward-related brain areas. 

In order to examine whether connectivity-based findings in PG patients are associated with behavioral measures, we explored the correlation between functional connectivity of the relevant networks and impulsiveness, symptom severity and smoking within the PG group. We found positive correlations between right middle frontal gyrus and right striatum connectivity and the nonplanning impulsiveness subscore and craving for gambling. In addition, the number of cigarettes per day positively correlated with the strengths of connectivity between right middle frontal seed and right striatum and with the strengths of connectivity between right ventral striatal seed and cerebellum. The positive correlations suggest that the alterations in functional connectivity are related not only to craving, but also to an indicator of the ability to plan for the future − for example, orientation to present goals and pleasures − and substance use behavior such as smoking. While Reuter et al. [27] showed that ventral striatal and ventromedial prefrontal activity during obtaining monetary gain in PG predicted gambling severity measured by the KFG, we did not find any correlation between KFG and G-SAS scores and alterations in functional connectivity between PFC and striatum. Thus, the observed changes in functional connectivity might reflect underlying mechanisms that increase the probability of developing gambling behavior rather than the symptom severity of PG itself.

The seed regions used here for the functional connectivity analysis were lateralized to the right hemisphere. This is due to fact that they were based on the results of our previous VBM study [49] showing a significant difference in local gray matter volume centered in right PFC and right striatum between PG patients versus matched controls. The right lateralization is consistent with previous evidence showing that the prefrontal executive functions, such as inhibitory control, are mainly situated in the right hemisphere [61-63]. Moreover, the involvement of right PFC has also been shown for self-regulation [64-67]. With respect to the reward system, imaging studies on PG reported right lateralized changes during reward processing: Alterations only in right ventral striatum have been found in response to gambling stimuli [29] as well as during the processing of monetary reward [27]. 

As PG patients were not abstinent nor in therapy, the current study is limited in its generalizability. Comparison to other studies on substance dependence is difficult, as they have been largely performed on patients in an abstinent state [39,45]. In addition, the data acquired do not allow for the investigation of causal relationships between the connectivity networks [68], which would otherwise provide further understanding of the directional interaction between PFC and mesolimbic reward system. 

In conclusion, our results demonstrate alterations in functional connectivity in PG with increased connectivity between regions of the reward system and the PFC, similar to those reported in substance use disorders. An imbalance between prefrontal function and the mesolimbic reward system in PG, and more generally in addiction, might benefit from both biological and psychotherapeutic interventions, such as a specialized cognitive behavioral [69] or euthymic therapy [70] that focus on normalizing network interactions related to reward processing.

## Supporting Information

File S1 **Supplementary Methods and Supplementary Results.**
(PDF)

Figure S1 **Signal loss in orbitofrontal cortex / ventral striatum** : One control subject (1002) and five PG patients (2011, 2019, 2044, 2048, 2061) had less than 50 % of voxels with signal within the right ventral striatal seed (green). Exemplary, subject 1001 had signal in every voxel within the seed.
(TIF)

Figure S2 **Functional connectivity of right middle frontal seed is not driven by gray matter volume differences** : Functional connectivity analysis with and without gray matter as covariate results in almost the same significant voxels (overlap shown in yellow). Voxels demonstrating significant correlations for the analysis with gray matter as covariate are shown in red. Voxels demonstrating significant correlations for the analysis without any covariate are shown in blue. Seed is depicted in green. A) Significantly positive correlations across both groups, B) significantly negative correlations across both groups, C) and D) group contrasts for significant correlations. N_controls_ = 19, N_PGsubjects_ = 19.
(TIF)

Figure S3 **Functional connectivity of right ventral striatal seed is not driven by gray matter volume differences** : Functional connectivity analysis with and without gray matter as covariate results in almost the same significant voxels (overlap shown in yellow). Voxels demonstrating significant correlations for the analysis with gray matter as covariate are shown in red. Voxels demonstrating significant correlations for the analysis without any covariate are shown in blue. Seed is depicted in green. A) Significantly positive correlations across both groups, B) significantly negative correlations across both groups, C) group contrast for significant correlations: PG patients > controls. Please note that the group contrast controls > PG patients was not significant. N_controls_ = 18, N_PGsubjects_ = 14.
(TIF)

Table S1 **Brain regions exhibiting significant connectivity across both groups and for the group contrasts in the functional connectivity analysis without gray matter regression.**
(PDF)
