# Correction: Increased Functional Connectivity between Prefrontal Cortex and Reward System in Pathological Gambling

**DOI:** 10.1371/journal.pone.0134179

**Published:** 2015-07-21

**Authors:** The PLOS ONE Staff

In the “Connectivity from the right middle frontal gyrus (N_controls_ = 19, N_PGpatients_ = 19)” subsection of the Results, the sentence, “Connectivity from the right ventral striatum (N_controls_ = 18, N_PGpatients_ = 14),” should appear as the heading of a new subsection. The publisher apologizes for the error.
