# Supplementary material for: Increased Functional Connectivity between Prefrontal Cortex and Reward System in Pathological Gambling
Source: PLoS One. 2013 Dec 19;8(12):e84565. doi: 10.1371/journal.pone.0084565 (PMC3868704; doi:10.1371/journal.pone.0084565)
Supplement: File S1 — Supplementary Methods and Supplementary Results. (PDF) [file pone.0084565.s001.pdf]

Supplementary Methods

Participants of the fMRI study

Twenty patients with symptoms of pathological gambling (PG), 15 alcohol-dependent patients and 21 healthy controls (all males) participated in an fMRI study at the Charité − Universitätsmedizin Berlin. For the resting-state functional connectivity analysis, we focused on the PG patients compared to healthy controls. One of the PG patients terminated the fMRI procedure before the resting-state measurement took place, and was thus excluded from the study. One of the control subjects had to be excluded due to technical problems during the functional measurements. In order to match the groups for age, another control subject was excluded due to his high age of 60 years (more than 2 SD away from the PG mean age, $M_{PG} = 32.79$). Thus, 19 PG patients and 19 controls remained for the resting-state analysis.

In addition, we had to exclude 5 PG patients and 1 control subject from the functional connectivity analysis of the ventral striatal seed region due to lack of complete brain coverage in that area (Figure S1).

Procedure

Upon arrival to the laboratory, subjects provided informed consent, and completed a questionnaire and instruction session. All subjects underwent a 75 minutes MRI session. Resting-state fMRI was acquired at the end of the MRI session after a T1 weighted structural measurement and three experimental tasks.

Supplementary Results

Functional connectivity analysis without gray matter regressions resulted in almost the same significant voxels both for the right middle frontal gyrus seed region and the right ventral striatal seed region (Figure S2, Figure S3, Table S1).
