# Supplementary figures and images for: Increased Functional Connectivity between Prefrontal Cortex and Reward System in Pathological Gambling
Source: PLoS One. 2013 Dec 19;8(12):e84565. doi: 10.1371/journal.pone.0084565 (PMC3868704; doi:10.1371/journal.pone.0084565)

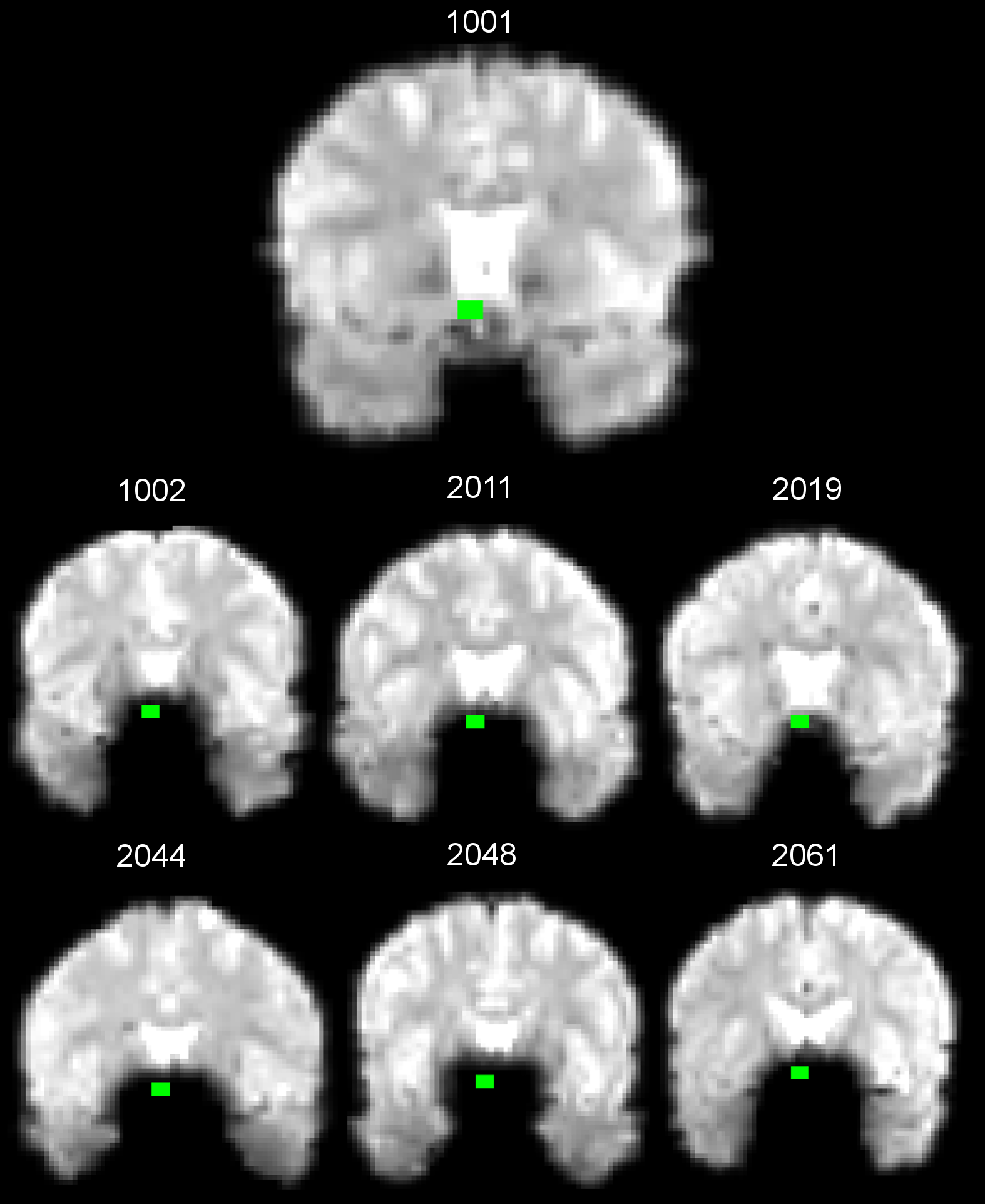

Supplement: Figure S1 — Signal loss in orbitofrontal cortex / ventral striatum : One control subject (1002) and five PG patients (2011, 2019, 2044, 2048, 2061) had less than 50 % of voxels with signal within the right ventral striatal seed (green). Exemplary, subject 1001 had signal in every voxel within the seed. (TIF) [file pone.0084565.s002.tif]

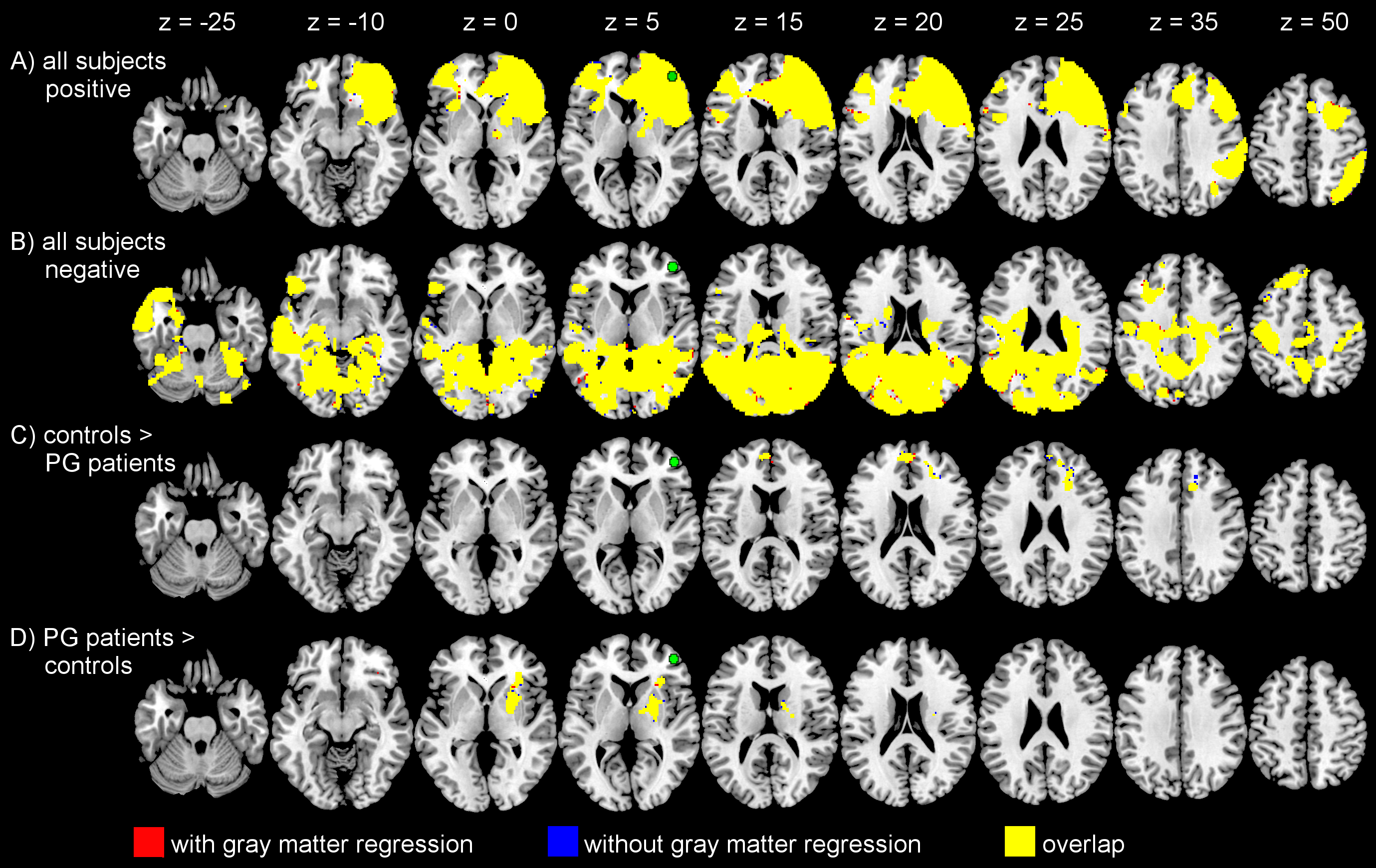

Supplement: Figure S2 — Functional connectivity of right middle frontal seed is not driven by gray matter volume differences : Functional connectivity analysis with and without gray matter as covariate results in almost the same significant voxels (overlap shown in yellow). Voxels demonstrating significant correlations for the analysis with gray matter as covariate are shown in red. Voxels demonstrating significant correlations for the analysis without any covariate are shown in blue. Seed is depicted in green. A) Significantly positive correlations across both groups, B) significantly negative correlations across both groups, C) and D) group contrasts for significant correlations. Ncontrols = 19, NPGsubjects = 19. (TIF) [file pone.0084565.s003.tif]

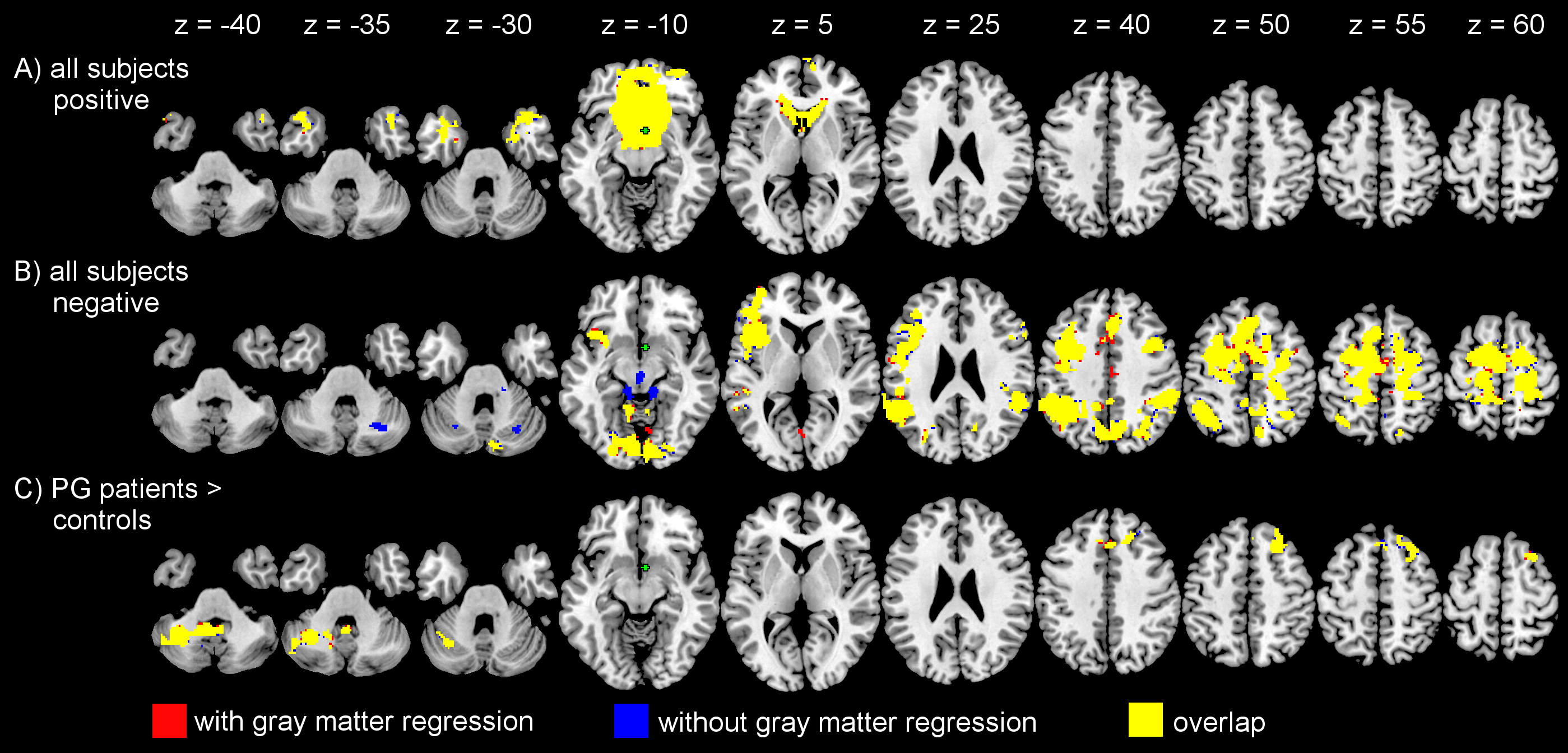

Supplement: Figure S3 — Functional connectivity of right ventral striatal seed is not driven by gray matter volume differences : Functional connectivity analysis with and without gray matter as covariate results in almost the same significant voxels (overlap shown in yellow). Voxels demonstrating significant correlations for the analysis with gray matter as covariate are shown in red. Voxels demonstrating significant correlations for the analysis without any covariate are shown in blue. Seed is depicted in green. A) Significantly positive correlations across both groups, B) significantly negative correlations across both groups, C) group contrast for significant correlations: PG patients > controls. Please note that the group contrast controls > PG patients was not significant. Ncontrols = 18, NPGsubjects = 14. (TIF) [file pone.0084565.s004.tif]
