# Supplementary material for: Increased Functional Connectivity between Prefrontal Cortex and Reward System in Pathological Gambling
Source: PLoS One. 2013 Dec 19;8(12):e84565. doi: 10.1371/journal.pone.0084565 (PMC3868704; doi:10.1371/journal.pone.0084565)
Supplement: Table S1 — Brain regions exhibiting significant connectivity across both groups and for the group contrasts in the functional connectivity analysis without gray matter regression. (PDF) [file pone.0084565.s005.pdf]

Table S1.

Seed	Contrast	Anatomical region	Side	Cluster-level *p* value (corrected)	Cluster size (voxels)	Voxel-level *z* value	MNI coordinates at peak voxel		
							x	y	z
Right middle frontal gyrus	mean positive	frontal pole	R	< .0001	26288	10.7	46	48	10
	mean negative	posterior cingulate gyrus	L	< .0001	50944	7.38	-14	-50	32
	PG < controls	cingulate gyrus	R	.0078	567	3.67	18	20	30
	PG > controls	putamen	R	.0022	685	3.46	26	0	-2
Right ventral striatum	mean positive	nucleus accumbens	R	< .0001	9132	9.2	4	6	-8
	mean negative	precentral gyrus	L	< .0001	18107	5.34	-50	2	20
		lingual gyrus	L	< .0001	2974	4.68	-8	-80	-12
	PG < controls			not significant					
	PG > controls	cerebellum	L	.0024	672	4.4	-32	-52	-38
		superior frontal gyrus	R	.0065	581	3.92	26	26	50
